# Reframing macrophage polarization through cholesterol efflux: an organelle-coupled immunometabolic model

**DOI:** 10.3389/fimmu.2026.1876507

**Published:** 2026-07-22

**Authors:** Ya Zou, Yun Lu, Lu Zhou, Qinchuan Li, Hua Wei, Yan Zhou, Shibo Lin, Xirui Guo, Shihao Yan, Hongju Wang, Fangqing Xie, Chun Liu

**Affiliations:** Department of Clinical Pharmacology, Sichuan University affiliated Chengdu Second People's Hospital, Chengdu Second People's Hospital, West China School of Medicine, Sichuan University, Chengdu, China

**Keywords:** atherosclerosis, cholesterol efflux, diabetic kidney disease, immunometabolism, macrophage polarization, mitochondria-lysosome axis, organelle homeostasis

## Abstract

Macrophages reside at the interface of immunity and metabolism, where their functional states are traditionally described by the M1/M2 polarization paradigm. However, this binary framework fails to capture the dynamic integration between inflammatory signaling and lipid metabolism that underlies macrophage behavior in chronic diseases. A central unresolved question is why macrophages, under sustained inflammatory and metabolic stress, progressively lose their capacity to maintain cholesterol homeostasis. Here, we propose a conceptual framework that is not merely a reinterpretation of existing data but a testable model: the macrophage polarization–efflux coupling axis, in which macrophage functional states are governed by the coordinated integration of lipid metabolism and organellar homeostasis, particularly the mitochondria–lysosome axis. Current evidence suggests that impaired cholesterol efflux may function as an active driver, rather than merely a downstream consequence, of macrophage dysfunction, based on evidence showing that genetic or pharmacological restoration of efflux actively repolarizes inflammatory macrophages toward a resolving phenotype. Lipid accumulation is reframed as a consequence of system-level failure arising from mismatched mitochondrial energy metabolism and lysosomal processing capacity. We further synthesize evidence demonstrating how transcriptional regulators, microRNA networks, epigenetic memory, and post-translational modifications converge to stabilize this dysfunctional state across diseases such as atherosclerosis and diabetic kidney disease. Importantly, emerging therapeutic strategies that restore organellar integrity and metabolic coordination show greater promise than approaches solely targeting inflammatory polarization. This integrative perspective shifts the focus from static phenotypic classification toward dynamic metabolic–organelle coupling, providing a unifying framework for understanding macrophage dysfunction and identifying novel therapeutic opportunities.

## 1 Introduction: from dichotomy to coupling – reframing macrophage functional states

The macrophage, a versatile innate immune cell, resides at the critical intersection of host defense and tissue homeostasis (1). Traditionally, this plasticity has been conceptualized through the M1/M2 polarization paradigm, where pro-inflammatory M1 macrophages and anti-inflammatory M2 macrophages represent opposing activation states (2–5). While useful for *in vitro* experiments, this dichotomy is increasingly recognized as an oversimplification that fails to capture the metabolic and environmental complexity observed *in vivo* (6, 7).

A critical limitation of the traditional framework is its implicit assumption that inflammatory and metabolic pathways operate in parallel. Here, we argue that they are, in fact, mechanistically inseparable. In physiological and pathological contexts, macrophages are continuously exposed to lipid species, cytokines, and metabolic stressors that simultaneously influence both their inflammatory phenotype and metabolic capacity (8–13). In particular, macrophages in chronic diseases such as atherosclerosis and diabetic kidney disease exhibit a progressive loss of cholesterol efflux capacity, leading to lipid accumulation, foam cell formation, and sustained inflammation (14–16).

This raises a central question that the M1/M2 paradigm cannot answer: why do macrophages fail to maintain cholesterol homeostasis under chronic inflammatory stress?

**Figure 1:**
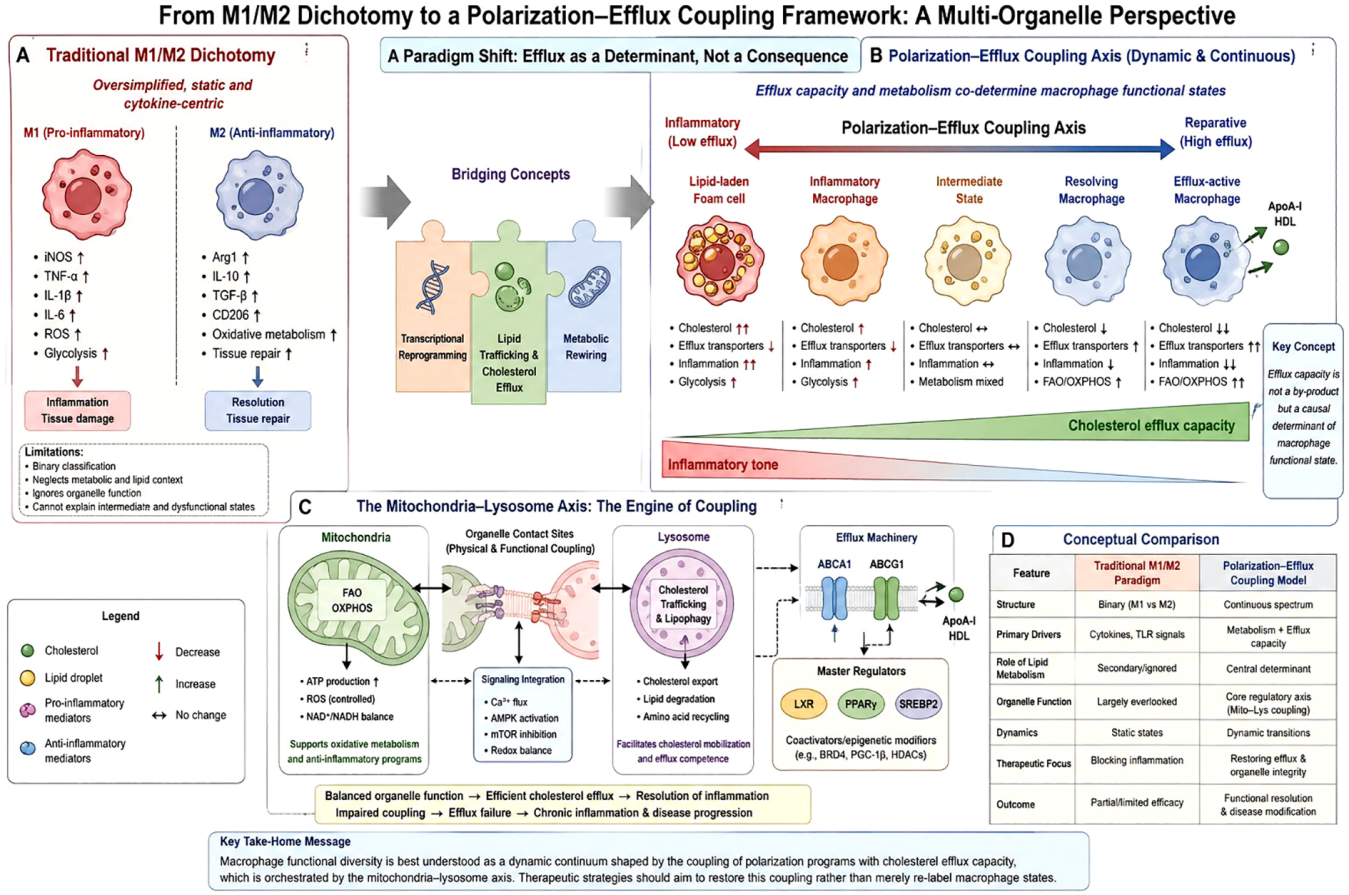
From M1/M2 dichotomy to a polarization-efflux coupling framework: a multi-organelle perspective.

To address this gap, we propose a fundamental reframing. As illustrated in **Figure 1**, rather than viewing M1 and M2 as fixed opposing states, we advance the macrophage polarization–efflux coupling axis as a unifying framework. Within this model, the mitochondria–lysosome axis serves as the central hub integrating lipid processing, energy metabolism, and inflammatory signaling (12, 17). The M1 polarization program often coincides with metabolic reprogramming toward aerobic glycolysis and can suppress cholesterol efflux pathways, promoting intracellular lipid accumulation (18). On the other hand, M2-polarizing signals frequently upregulate oxidative metabolism and enhance cholesterol efflux transporter expression (4). The dysregulation of this delicate balance is a cornerstone of chronic inflammatory metabolic diseases (19). In atherosclerosis, the inability of macrophages within the plaque to effectively efflux cholesterol leads to foam cell formation (14, 15). Similarly, in diabetic kidney disease, impaired macrophage cholesterol efflux under hyperglycemic conditions contributes to renal inflammation and fibrosis (5, 16).

Crucially, restoring healthy macrophage function hinges on re-establishing lipid metabolic homeostasis and organellar integrity, rather than simply pushing cells from one polarized label to another (20). This insight distinguishes our framework from prior reviews: we do not merely describe the association between polarization and metabolism; we argue that efflux capacity is a primary determinant of functional outcome. This review aims to synthesize current knowledge on this intricate interplay, exploring its transcriptional, post-transcriptional, and epigenetic regulation, its pathophysiological consequences, and the emerging therapeutic strategies designed to restore this critical balance (21).

## 2 Fundamental interplay between macrophage polarization and cholesterol metabolism: beyond the M1/M2 dichotomy

### 2.1 The metabolic signatures of macrophage functional states: a causality debate

A growing body of evidence suggests that the metabolic profiles of M1 and M2 macrophages are not merely correlates of their functional states but may actively reinforce them through specific biochemical constraints.

The classical pro-inflammatory “M1-like” activation, often in response to toll-like receptor (TLR) ligands like LPS, is associated with a shift toward glycolysis and the pentose phosphate pathway, supporting rapid ATP generation and the production of biosynthetic precursors for inflammatory mediators (2). This metabolic rewiring often comes at the expense of pathways involved in lipid catabolism and efflux (18). In this state, macrophages mount a highly energy-demanding defense response (22). Central to this metabolic switch is the upregulation of glycolytic enzymes and suppression of mitochondrial oxidative phosphorylation, which limits the cell’s capacity for fatty acid oxidation (23). For instance, IFN-γ downregulates ABCA1 expression via post-transcriptional destabilization of ABCA1 mRNA, impairing cholesterol efflux to apoA-I and promoting cholesteryl ester accumulation (24). Furthermore, M1-associated signals can upregulate ACAT1 activity, promoting cholesterol esterification and lipid droplet formation. Cholesterol crystals directly drive M1 polarization in primary human macrophages, upregulating GLUT1, HK2, HIF1α, and PFKFB3 while suppressing mitochondrial respiration, and this process is mediated by PKM2 nuclear translocation and reversed by PKM2 tetramerization or glycolytic inhibition (25). The NLRP3 inflammasome is activated by cholesterol crystals and further amplifies inflammation (26).

Conversely, the anti-inflammatory ‘M2-like’ polarization is linked to enhanced mitochondrial oxidative phosphorylation and FAO (23, 27). Key FAO enzymes such as CPT1A, CROT, and HADHB are upregulated in M2 cells (28, 29). The M2 phenotype also exhibits higher basal cholesterol efflux capacity via ABCA1/ABCG1 (30), and efficient efflux itself reduces lipid raft formation and dampens TLR signaling (31).LXR activation, which promotes cholesterol efflux, also upregulates genes involved in lysosomal cholesterol trafficking (NPC1, NPC2) and reduces mitochondrial oxidative stress, thereby reinforcing the M2 metabolic program (32). The transcription factor TFEB appears to play a central role in coordinating these organellar responses (33).

While these observations support the idea that metabolic configuration actively shapes inflammatory output, it is important to note that much of this evidence remains correlational. Definitive causal demonstrations—showing, for example, that forced metabolic reprogramming directly reverses an inflammatory phenotype *in vivo*—are still limited and represent a priority for future research. Furthermore, some well-established relationships in murine systems have proven to be more complex in humans. For instance, the dependency on FAO appears context-dependent and may differ between species, as human M2 polarization does not strictly require this pathway (34). Similarly, the correlation between ABCA1 expression and functional efflux capacity is imperfect; some studies show high ABCA1 mRNA without corresponding efflux activity, suggesting that post-transcriptional control can dominate in certain contexts (30, 35). Nevertheless, mitochondrial-lysosome axis integrity remains critical across species, as its disruption consistently impairs M2 maintenance (36).

### 2.2 The mitochondria-lysosome axis as a central hub

A key insight emerging from recent studies is that the functional state of a macrophage is critically dependent on the integrity and coordination of two major organellar systems: mitochondria and lysosomes (17). This has led to the concept of a “mitochondria-lysosome axis” that may serve as a central hub integrating lipid metabolism and inflammatory signaling, where metabolic signals are translated into polarization decisions. Importantly, this coordination extends beyond pairwise interactions: recent evidence reveals that macrophages organize mitochondria, lysosomes, lipid droplets, and the ER into dynamic “functional multi-organelle units” that collectively control inflammatory lipid metabolism, suggesting that the integrity of the entire organelle network, rather than any single compartment, dictates cellular outcomes.

In the M1-like state, the metabolic program comes with a cost: mitochondrial dysfunction (e.g., decreased membrane potential, increased fragmentation) and suppression of fatty acid oxidation (37). In diabetic conditions, mitochondrial ROS directly induce lysosomal dysfunction, impairing autophagic flux and contributing to M1 polarization (17). More importantly, the capacity of lysosomes to process internalized lipids (such as oxidized low-density lipoprotein, oxLDL) is diminished (38), characterized by reduced acidification and decreased activity of lysosomal acid lipase (LAL). This leads to accumulation of unesterified cholesterol and cholesteryl esters, ultimately driving foam cell formation (39, 40). LXR activation enhances lysosomal cholesterol trafficking by inducing NPC1 and NPC2 expression, coupling lysosomal function with cholesterol efflux (32). Viewed from this perspective, lipid accumulation in the M1-like state can be understood as collateral damage—a consequence of the mismatch between mitochondrial energy metabolism and lysosomal processing capacity (41).

Conversely, during M2-like polarization, the mitochondria-lysosome axis appears to operate in a more coordinated and efficient manner. M2-polarizing signals (e.g., IL-4) upregulate PPARγ and LXRα, which not only increase the expression of cholesterol efflux transporters but also enhance lysosomal hydrolase activity, thereby improving the processing efficiency of ingested lipids derived from apoptotic cells or lipoproteins (35, 42). A functional M2-like state may therefore reflect the efficient cooperation of the mitochondria-lysosome axis, maintaining a dynamic balance of lipid “influx-processing-efflux.”

Taken together, the mitochondria-lysosome axis offers a parsimonious explanation for how metabolic stress can lead to polarized dysfunction. It remains to be determined, however, whether primary lysosomal defects can initiate M1 polarization independently of mitochondrial signals—a hypothesis that will require direct experimental testing.

### 2.3 Lipid accumulation as collateral damage, not an active choice

It has often been assumed that lipid-laden macrophages are actively pro-inflammatory. An alternative perspective, however, is that lipid accumulation may represent an unintended consequence—a form of system failure—rather than an adaptive feature of the M1 program.

This perspective offers a different way of understanding foam cell formation. In the M1-like state, the cell prioritizes rapid glycolytic energy production and inflammatory mediator synthesis, apparently at the expense of maintaining robust lipid handling machinery (25, 43). The resulting accumulation of cholesterol esters can therefore be viewed as a side effect of this metabolic prioritization, rather than an actively selected feature of the M1 program (18). TLR4 signaling through SREBP-1a upregulates fatty acid synthesis and cholesterol esterification without inducing ABCA1, resulting in a relative efflux deficiency that favors lipid accumulation (44). The resulting accumulation of cholesterol esters can therefore be viewed as a side effect of this metabolic prioritization, rather than an actively selected feature of the M1 program.

If lipid accumulation is indeed best understood as collateral damage rather than an active pathogenic driver, then this reframing carries potential therapeutic implications. Simply attempting to shift cells from an M1 to an M2 phenotype may be insufficient. Instead, therapies that restore the functional capacity of the mitochondria-lysosome axis—enabling macrophages to handle lipid loads regardless of their polarization state—could prove more effective.

### 2.4 The vicious cycle: failed efflux reinforces inflammation

**Figure 2:**
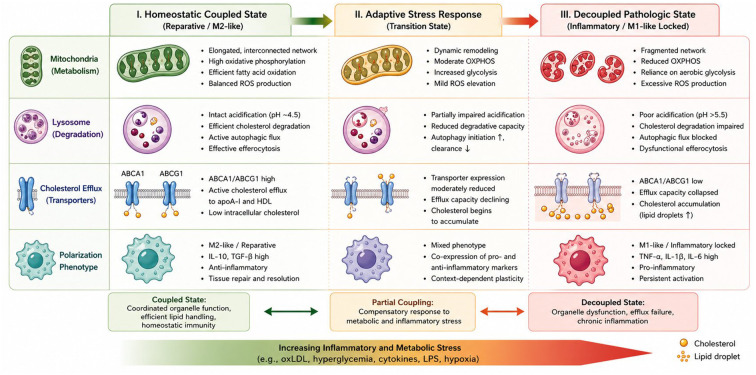
These changes depict a progressive continuum linking organelle dysfunction to altered macrophage lipid handling and phenotypic plasticity. Schematic illustration of the progressive transition of macrophages from a homeostatic coupled state to a decoupled pathological state under increasing metabolic and inflammatory stress. In the homeostatic coupled state, coordinated mitochondrial oxidative metabolism, lysosomal acidification, and ABCA1/ABCG1-mediated cholesterol efflux support reparative polarization and efficient lipid handling. Under adaptive stress conditions, partial disruption of organelle coordination induces metabolic remodeling, reduced lysosomal degradative capacity, and declining cholesterol efflux, resulting in a transitional mixed-polarization phenotype. Sustained exposure to pathological stimuli, including oxidized lipids, hyperglycemia, cytokines, hypoxia, and endotoxin signals, drives progression toward a decoupled pathological state characterized by mitochondrial fragmentation, lysosomal dysfunction, impaired cholesterol transport, intracellular lipid accumulation, and persistent pro-inflammatory activation. This dynamic continuum highlights polarization–efflux coupling as a central determinant of macrophage functional plasticity and immunometabolic homeostasis.

The relationship between efflux failure and inflammation may not be simply linear. Instead, evidence suggests that it can become circular—a self-reinforcing cycle that may help explain the chronicity of inflammatory diseases. The conceptual framework is illustrated in **Figure 2**.

In the microenvironment of atherosclerosis or obesity, macrophages are chronically exposed to mixed signals (e.g., oxLDL + IFN-γ) (45, 46). They appear to adopt a metabolic stress-adaptive state characterized by partial mitochondrial dysfunction, incomplete lysosomal lipid hydrolysis, and impaired efflux, ultimately leading to NLRP3 inflammasome activation (26). Cholesterol crystals directly activate the NLRP3 inflammasome in a manner dependent on mitochondrial ROS and lysosomal damage (26). ER stress further inhibits M2 polarization and cholesterol efflux while promoting inflammation (47, 48), involving UPR pathways (PERK, IRE1α, ATF6) that directly suppress LXRα expression (47).ER stress through the PERK/ATF6 axis downregulates LXRα, reducing ABCA1/ABCG1 expression and impairing efflux (48). A vicious cycle can therefore become established (12).

If this cycle is indeed central to disease progression, then interrupting any of its nodes could be beneficial. It is plausible, however, that restoring efflux capacity might be a particularly efficient intervention, as it could simultaneously reduce the inflammatory trigger (lipid accumulation) and help restore organellar homeostasis.

## 3 Molecular regulation of the coupling axis

### 3.1 Transcriptional integration: LXR, PPARγ, and SREBP as master switches

Nuclear receptors are increasingly recognized not simply as passive responders to lipid signals but as active integrators that continuously link metabolic load with transcriptional output to maintain cholesterol efflux capacity within a viable range.

LXR and PPARγ serve as central integrators in this process (49–51), promoting cholesterol efflux via ABCA1/ABCG1 while simultaneously suppressing pro-inflammatory gene expression (52, 53). LXR activation directly upregulates NPC1 and NPC2, enhancing lysosomal cholesterol trafficking to the plasma membrane and thereby promoting efflux at the expense of esterification. LXR activation also induces 15-LOX in M2-like macrophages, generating anti-inflammatory oxylipins such as 5,15-diHETE, further linking cholesterol sensing to resolution pathways (54). LXR activation directly alleviates lysosomal and mitochondrial lipid burden (55, 56). PPARγ activation promotes M2 polarization, enhances efflux, and improves mitochondrial FAO capacity and lysosomal function (53, 57). The CTRP12/miR-155-5p/LXRα axis exemplifies this integration (57). Other transcription factors, such as MafB, also promote both M2 polarization and cholesterol efflux (58), whereas M1-driving factors like IRF1 and STAT1 antagonize these pathways (59, 60). SUB1 promotes atherosclerosis by activating IRF1 transcription (61).

SREBPs represent another crucial family linking lipid biosynthesis to immune function (44, 62). SCAP, the escort protein for SREBP translocation, is essential for SREBP activation (63). Macrophage-specific SCAP deficiency impairs LPS-mediated induction of SREBP-1a, disrupting a downstream cascade involving cholesterol 25-hydroxylase and 25-hydroxycholesterol production (64). SREBP-1a is required for TLR4-induced lipogenesis and phagocytosis, and its deletion reduces both lipid synthesis and inflammatory activation (Lee et al., 2018). SCAP deficiency leads to reduced LXRα activation, diminished cholesterol efflux, and a shift toward pro-inflammatory M1 polarization in adipose tissue, exacerbating obesity and insulin resistance. The SREBP2 host gene encodes miR-33a, while SREBP1 encodes miR-33b in humans, creating a direct link between cholesterol biosynthesis regulation and post-transcriptional control of efflux (65).

One notable feature of this transcriptional network is its apparent redundancy, with multiple factors converging on the same efflux transporters. This observation raises the possibility that targeting downstream effectors (e.g., ABCA1 directly) might be more therapeutically tractable than modulating upstream regulators that have broad metabolic consequences.

It should be noted, however, that the therapeutic application of these insights faces practical challenges. For example, while LXR activation potently promotes efflux, it also causes hepatic steatosis and hypertriglyceridemia (66), indicating that systemic targeting is problematic. Similarly, PPARγ activation improves efflux but is associated with weight gain and fluid retention (67), suggesting that cell-specific targeting strategies may be needed.

### 3.2 Post-transcriptional control by microRNAs: fine-tuning vs. dominant control

MicroRNAs have emerged as important regulators of the polarization-efflux axis. While some miRNAs act as fine-tuners, others—particularly miR-33 and miR-34a—appear to exert more dominant control by simultaneously targeting multiple nodes within the network.

miR-33a/b repress cholesterol efflux via ABCA1/ABCG1 and also inhibit FAO genes (CROT, CPT1A, HADHB, PRKAA1) (28, 29). Conversely, anti-miR-33 enhances mitochondrial respiration and promotes M2 polarization (68, 69). miR-34a targets ABCA1/ABCG1 and LXRα, and its ablation inhibits atherosclerosis (70). Other miRNAs play distinct roles: miR-223 promotes efflux by targeting the transcription factor Sp3 at the translational level, and miR-223 deficiency in macrophages increases pro-inflammatory cytokine production and exacerbates atherosclerosis (71); miR-155-5p inhibits LXRα (72); miR-21 deficiency reduces ABCG1 stability, worsening plaque necrosis and inflammation (73); and miR-205-5p promotes unstable plaque formation (74).

An interesting feature of this regulatory landscape is the participation of these miRNAs in feedback loops with the very transcription factors they regulate. For example, miR-155 and miR-33 can target MafB—a promoter of M2 polarization and efflux—creating a double-negative feedback loop (75). The SREBP2-miR-33-ABCA1 axis forms another such loop: when cholesterol levels are low, SREBP2 is activated to promote cholesterol synthesis while simultaneously upregulating miR-33, which suppresses ABCA1-mediated efflux, thereby preventing further cholesterol depletion (76).

The presence of such embedded feedback loops suggests that the system may have evolved to resist perturbation. This could help explain why single-target interventions often prove unsuccessful, and why strategies that target miRNAs—which can affect multiple nodes simultaneously—have shown unusual efficacy in preclinical studies. Key molecular regulators are summarized in **Table 1**.

**Table 1:** Key regulators of the macrophage polarization–efflux coupling axis.

Molecule/pathway	Type	Key targets/mechanisms	Impact on polarization-efflux axis
LXRα/β	Nuclear receptor	ABCA1, ABCG1, MyD88 (alternative splicing)	Promotes efflux + Anti-inflammatory
PPARγ	Nuclear receptor	ABCA1, ABCG1, CD36, FAO genes (CPT1A, CROT, HADHB)	Promotes M2 + Enhances efflux + Improves mitochondrial function
SREBP2/miR-33a	Transcription factor/miRNA	ABCA1, ABCG1, CPT1A, CROT, HADHB	Inhibits efflux + Suppresses FAO → Promotes M1
miR-34a	miRNA	ABCA1, ABCG1, LXRα	Inhibits efflux + Promotes foam cell formation
miR-223	miRNA	Sp3 (transcription factor)	Promotes efflux + Anti-inflammatory
miR-155-5p	miRNA	LXRα	Inhibits efflux + Pro-inflammatory
UBE3A	E3 ubiquitin ligase	ABCA1 (proteasomal degradation)	Accelerates efflux failure
PAK1	Kinase	PPARγ (suppresses expression)	Promotes M1 + Foam cell formation
AMPK/mTORC1	Energy sensor	Autophagy, mitophagy, mitochondrial biogenesis	Integrates metabolism and polarization

### 3.3 Organelle-dependent signaling nodes: when organelles become signaling platforms

Mitochondria and lysosomes are increasingly appreciated not merely as passive sites of metabolic reactions but as active signaling platforms that communicate their functional status to the nucleus and the inflammatory machinery.

Cellular stress pathways—including mitochondrial ROS production, ER stress, and NLRP3 inflammasome activation—have been shown to modulate macrophage function (17, 47). Mitochondrial ROS directly activate the NLRP3 inflammasome by promoting its oligomerization and ASC speck formation, a process amplified by cholesterol crystals (26). In diabetic conditions, mitochondrial ROS-induced lysosomal dysfunction impairs autophagic flux and contributes to M1 macrophage polarization (17).ER stress through the PERK/ATF6 pathway suppresses LXRα expression, thereby reducing ABCA1/ABCG1 and promoting foam cell formation.

**Figure 3:**
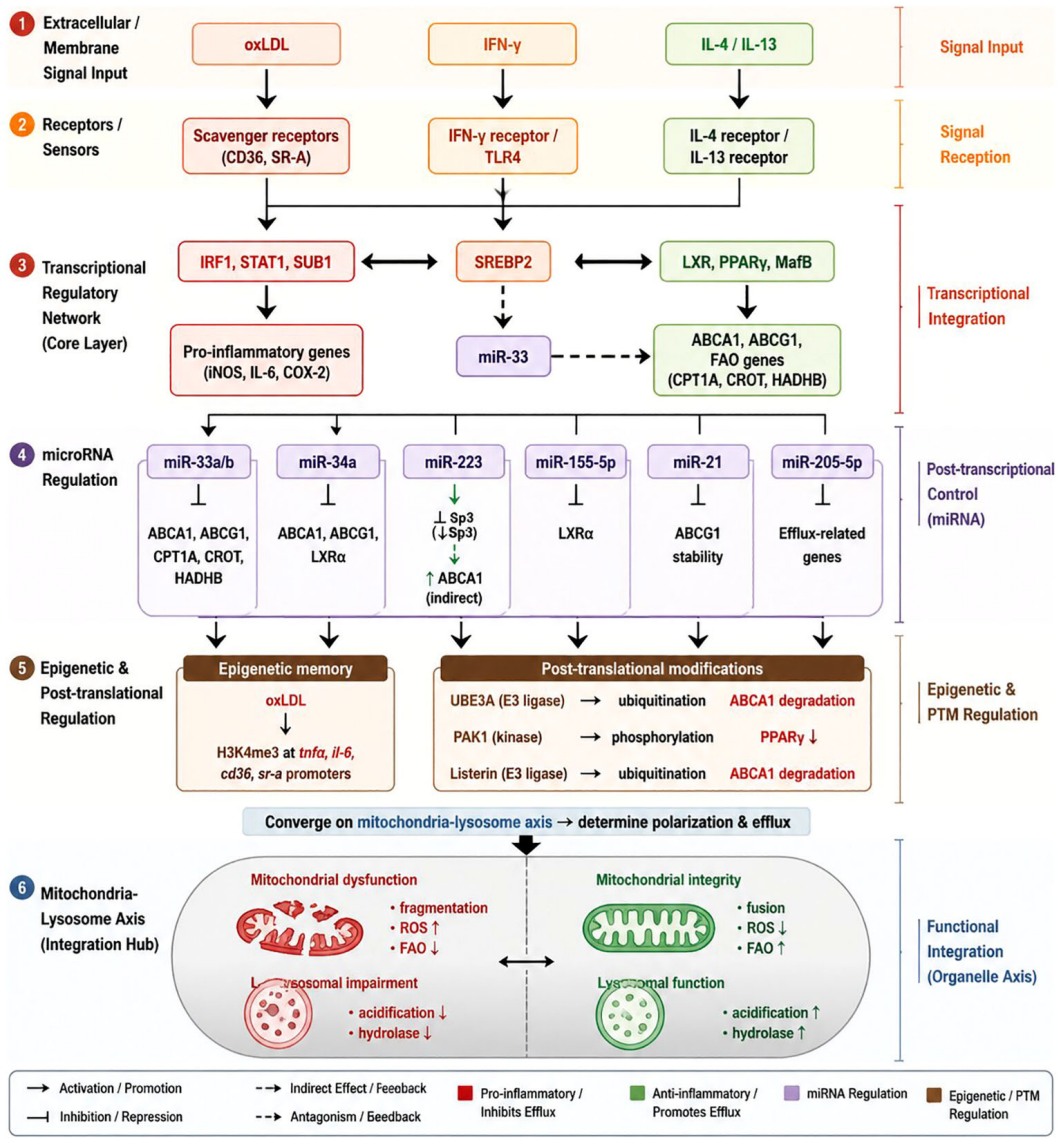
Molecular regulation of the polarization–efflux coupling axis. The diagram illustrates the multilayered network integrating extracellular signals, transcriptional regulators, microRNAs, epigenetic modifications, and post-translational modifications that converge on the mitochondria-lysosome axis to control macrophage polarization and cholesterol efflux. Top layer: Extracellular stimuli (oxLDL, IFN-γ, IL-4/IL-13) engage specific receptors. Second layer: Downstream transcription factors—pro-inflammatory (IRF1, STAT1, SUB1) and pro-resolving/efflux-promoting (LXR, PPARγ, MafB)—orchestrate opposing gene programs. SREBP2 links lipid synthesis to miR-33 expression. Third layer: microRNAs (miR-33a/b, miR-34a, miR-223, miR-155-5p, miR-21, miR-205-5p) fine-tune the axis by targeting ABCA1/ABCG1, FAO enzymes (CPT1A, CROT, HADHB), and LXRα. Fourth layer: Epigenetic reprogramming (oxLDL-induced H3K4me3) and post-translational modifications (UBE3A- and Listerin-mediated ubiquitination of ABCA1, PAK1-mediated phosphorylation of PPARγ) stabilize dysfunctional states. All layers ultimately converge on the mitochondria-lysosome axis, where the balance between organellar dysfunction (left) and integrity (right) dictates the net outcome of polarization and efflux capacity.

This organelle-to-nucleus signaling represents a relatively underexplored therapeutic frontier. If organellar dysfunction is both a cause and a consequence of inflammation, then strategies aimed at stabilizing organelle integrity might break the pathological cycle more effectively than blocking individual cytokines. The multilayered molecular network is summarized in **Figure 3**.

## 4 Epigenetic memory and functional lock-in: why macrophages cannot reset

An important question in the field is why dysfunctional macrophage states persist in chronic disease even when the original polarizing stimuli are no longer present. A growing body of evidence suggests that this may be explained not by sustained exposure to polarizing signals but rather by epigenetic memory that locks in metabolic dysfunction after the initial trigger has been removed.

Chronic exposure to oxidized lipids has been shown to induce a phenomenon known as trained immunity (77, 78), stabilizing a pro-inflammatory, lipid-retentive phenotype (79). Brief exposure of human monocytes to a low concentration of oxLDL induces long-lasting trimethylation of histone 3 lysine 4 (H3K4me3) at the promoters of pro-inflammatory genes tnfα,il–6 and genes involved in lipid uptake cd36,sr–a. This effect is completely prevented by inhibition of histone methyltransferases, underscoring the central role of epigenetic modifications (78). Such mechanisms may help explain the persistent low-grade vascular inflammation observed in atherosclerosis, even after the removal of acute triggers (79). Together, epigenetic modifications and feedback loops appear to render macrophage dysfunction resistant to reversal (80, 81).

Post-translational modifications provide an additional layer of rapid and dynamic control over the polarization-efflux axis (82). The stability of the crucial efflux transporter ABCA1 is tightly regulated by the ubiquitin-proteasome system. In lipid-loaded macrophages within demyelinating brain disorders, accumulation of myelin-derived lipids promotes the activity of the E3 ubiquitin ligase UBE3A, which targets ABCA1 for proteasomal degradation (83). Another E3 ligase, Listerin, has also been shown to ubiquitinate ABCA1, and its deficiency improves cholesterol efflux and reduces atherosclerosis (84). Phosphorylation events play a role as well: the kinase PAK1 is upregulated in M1 macrophages and promotes pro-inflammatory activation and foam cell formation by suppressing PPARγ expression; PAK1 inhibition shifts cells toward an M2-like phenotype and improves cholesterol handling (85). Conversely, inhibition of PAK1 leads to a shift toward an M2-like phenotype and improved cholesterol handling.

One implication of these findings is that short-term interventions may have long-term consequences through epigenetic memory. It also raises the possibility that strategies aimed at erasing this memory—for example, via activation of histone demethylases or inhibition of HDACs—could prove more effective than chronic pathway blockade.

## 5 Disease contexts: collapse of the coupling axis

### 5.1 Atherosclerosis: when the axis fails

Atherosclerosis has been proposed as a prototypical disease in which the polarization-efflux coupling axis fails, with evidence suggesting that multiple nodes—transcriptional, post-transcriptional, epigenetic, and organellar—may be compromised.

**Figure 4:**
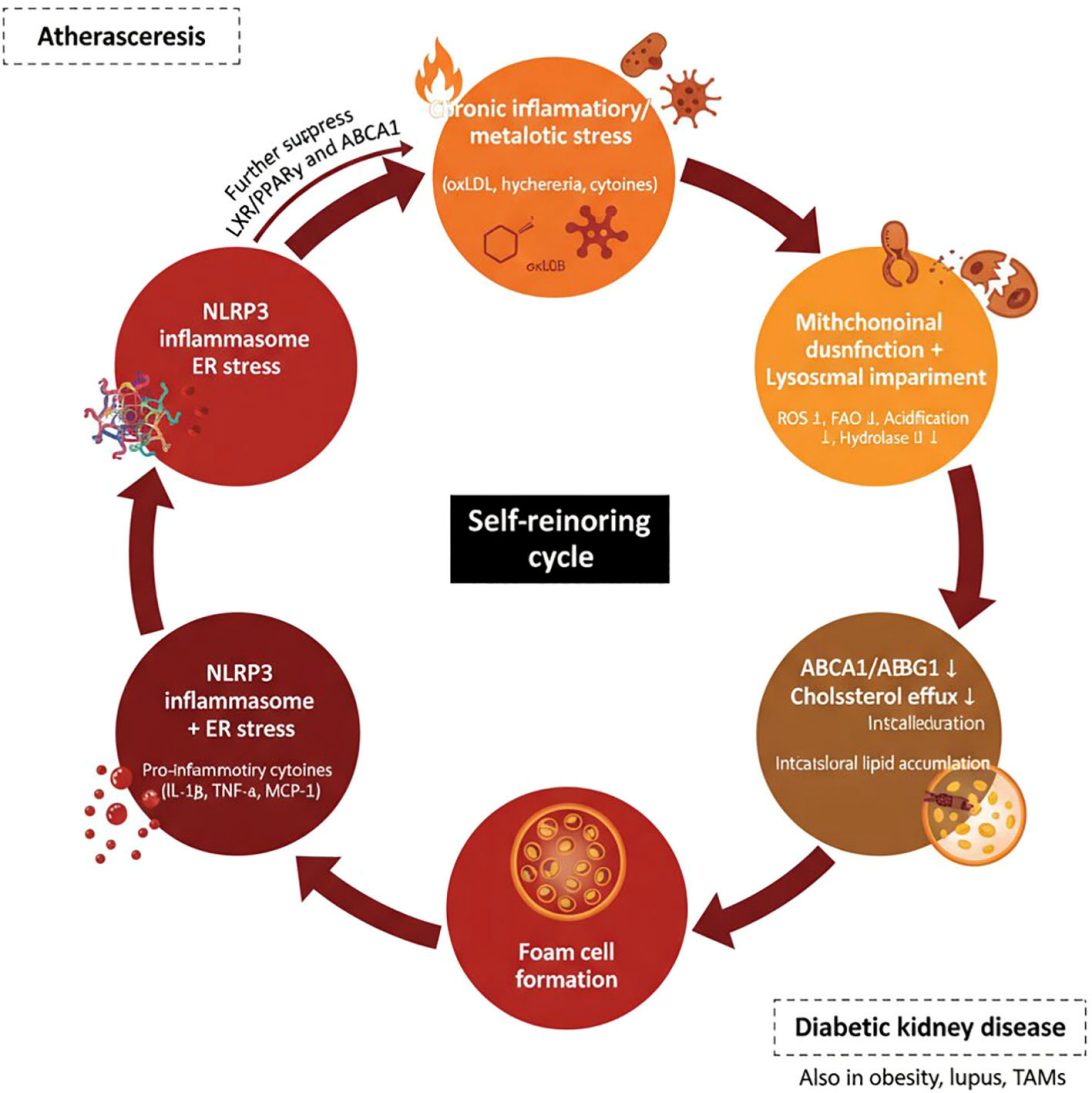
Vicious cycle of efflux failure and inflammation in atherosclerosis and diabetic kidney disease. The diagram illustrates a self-reinforcing cycle that drives disease progression. Chronic inflammatory or metabolic stress (oxLDL, hyperglycemia, cytokines) triggers mitochondrial dysfunction (increased ROS, decreased FAO) and lysosomal impairment (reduced acidification and hydrolase activity). These organellar defects suppress ABCA1/ABCG1 expression, leading to diminished cholesterol efflux and subsequent intracellular lipid accumulation. Lipid-laden macrophages transform into foam cells, a hallmark of early atherosclerotic lesions. Accumulated lipids also activate the NLRP3 inflammasome and induce ER stress, resulting in sustained production of pro-inflammatory cytokines (IL-1β, TNF-α, MCP-1). These inflammatory mediators further suppress LXR/PPARγ activity and ABCA1 expression, closing the loop and amplifying the cycle. This vicious cycle operates in atherosclerosis, diabetic kidney disease, and other chronic inflammatory conditions, and represents a central therapeutic target.

In the atherosclerotic microenvironment, macrophages are exposed to excessive lipid influx and inflammatory signals (86, 87). The normal homeostatic LXR-mediated efflux response appears to be overwhelmed (88). Inflammatory cytokines such as IFN-γ and TNF-α suppress ABCA1 expression and LXR activity (89). Mitochondrial dysfunction and impaired lysosomal acid lipase (LAL) activity further compromise the cell’s ability to process internalized lipid loads (90). The cholesterol transport protein ORP2 plays a protective role; its deficiency exacerbates foam cell formation, whereas overexpression promotes efflux and reduces atherosclerosis (15). oxLDL-induced epigenetic reprogramming entrenches a pro-inflammatory, lipid-retentive phenotype (91). The resulting accumulation of cholesterol esters transforms macrophages into foam cells, the defining cellular component of the fatty streak and early atherosclerotic plaque (92). These inflammatory foam cells secrete cytokines, chemokines, and matrix metalloproteinases (MMPs), promoting monocyte recruitment, smooth muscle cell modulation, and eventual plaque destabilization (92). Factors that disrupt efflux—such as miR-33, miR-34a, and UBE3A- and Listerin-mediated ABCA1 degradation (70, 83, 93, 94). Conversely, interventions that promote efflux (e.g., S1P1 signaling or Latexin deficiency) have been shown to be atheroprotective (95, 96). Given the multiplicity of nodes that appear to fail in this disease, it seems plausible that targeting any single pathway may be insufficient. Combination strategies—for example, LXR activation combined with Notch inhibition—have shown promise in preclinical studies by simultaneously addressing transcriptional and organellar defects (97). The self-reinforcing cycle is depicted in **Figure 4**.

### 5.2 Diabetic kidney disease: hyperglycemia as a dual insult

In diabetic kidney disease, macrophages face a uniquely challenging microenvironment. Hyperglycemia appears to damage both mitochondria and lysosomes directly, which may provide a “second hit” that helps explain why diabetic macrophages are particularly resistant to repolarization.

Under hyperglycemic conditions, advanced glycation end-products (AGEs) and other metabolic stressors suppress LXR and PPARγ signaling pathways, leading to downregulation of ABCA1 and ABCG1 and impaired cholesterol efflux, which in turn contributes to the formation of macrophage-derived foam cells within the kidney (16). Hyperglycemia also directly damages mitochondria (promoting fragmentation and reducing FAO capacity) while impairing lysosomal acidification and autophagic flux (98). Hypoxia in the diabetic kidney microenvironment, acting through HIF-1α, further suppresses fatty acid oxidation and promotes lipid synthesis, thereby exacerbating lipotoxicity (5). Of note, diabetic conditions also impair atherosclerosis regression after cholesterol lowering, an effect that appears to be partly mediated by hyperglycemia-induced monocytosis and sustained plaque inflammation (99). Interestingly, raising functional HDL levels in diabetic mice has been shown to overcome this impairment by suppressing myelopoiesis, reducing monocyte recruitment, and promoting plaque macrophage polarization toward the resolving M2 state (99).

Taken together, the convergence of hyperglycemic and inflammatory stress on the same organellar systems may help account for the accelerated and particularly refractory nature of macrophage dysfunction in the diabetic setting.

### 5.3 Cross-disease convergence: a unifying mechanism

**Figure 5:**
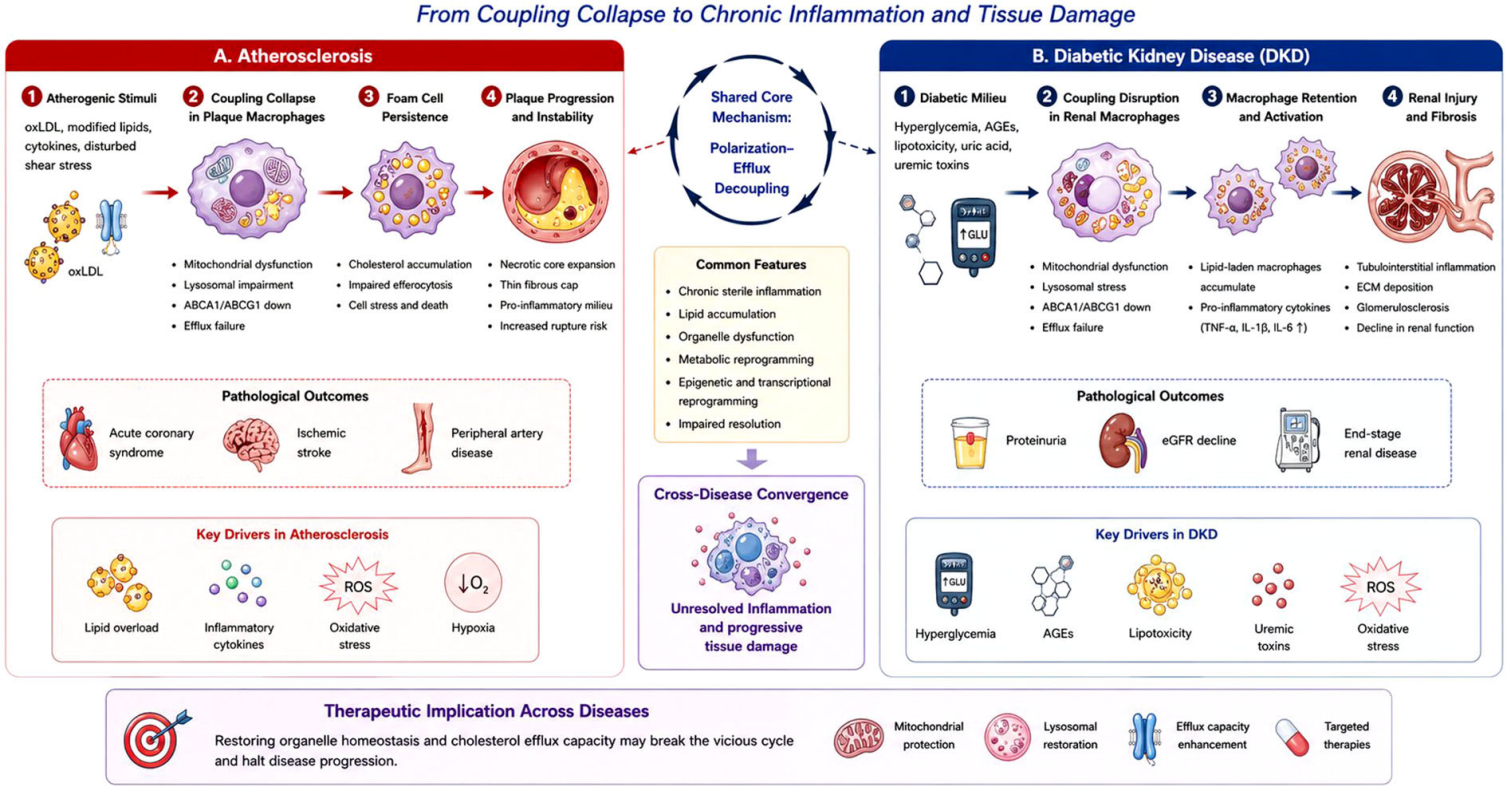
Disease-specific consequences of polarization–efflux. Decoupling. Schematic overview of the pathological consequences of macrophage polarization–efflux decoupling across distinct chronic inflammatory disease contexts. In atherosclerosis, exposure to oxidized lipids and inflammatory mediators disrupts mitochondrial and lysosomal homeostasis, suppresses cholesterol efflux pathways, and promotes foam cell persistence, necrotic core expansion, and plaque instability. In diabetic kidney disease, hyperglycemia, lipotoxic stress, and inflammatory signaling similarly impair organelle coordination and cholesterol handling, leading to macrophage retention, profibrotic activation, extracellular matrix accumulation, and progressive glomerular injury. Despite differences in tissue-specific microenvironments, both disease settings exhibit convergent features including intracellular lipid accumulation, sustained inflammatory activation, defective resolution responses, and amplification of chronic sterile inflammation. These shared pathological mechanisms highlight polarization–efflux decoupling as a common immunometabolic axis linking macrophage dysfunction to tissue injury progression.

Macrophage dysfunction across diverse chronic inflammatory diseases exhibits notable mechanistic convergence despite differences in tissue microenvironment and initiating pathological stimuli. In both atherosclerosis and diabetic kidney disease, sustained metabolic stress disrupts mitochondrial integrity, impairs lysosomal degradative capacity, and suppresses cholesterol efflux pathways, thereby promoting persistent inflammatory activation and defective resolution responses. These shared alterations suggest that macrophage polarization is closely intertwined with intracellular lipid handling and organelle homeostasis, forming an integrated immunometabolic axis that shapes disease progression. Collectively, these observations suggest that polarization–efflux decoupling represents a convergent immunometabolic mechanism across distinct chronic inflammatory diseases. The shared and disease-specific pathological consequences are summarized in **Figure 5**. This framework further supports the concept that restoring organelle coordination and cholesterol efflux capacity may represent a broadly applicable therapeutic strategy for interrupting maladaptive macrophage reprogramming across multiple disease contexts.

## 6 Therapeutic reprogramming of the axis

The therapeutic landscape has evolved considerably, with attention shifting from strategies that block individual inflammatory mediators toward approaches that aim to restore system-level metabolic–organellar coordination.

### 6.1 Transcriptional modulation

Pharmacological activation of nuclear receptors such as LXRs and PPARγ has been a direct approach (100). LXR agonists increase ABCA1/ABCG1 expression, stimulate cholesterol efflux, and exert anti-inflammatory effects, making them potent anti-atherosclerotic agents (100). However, their clinical development has been limited by on-target side effects including hepatic steatosis and hypertriglyceridemia (66). Combination therapy with a Notch inhibitor has been shown to enhance the anti-atherosclerotic effects of an LXR agonist while mitigating fatty liver development in mice, suggesting a viable strategy to overcome these limitations (97). PPARγ agonists (thiazolidinediones) also promote M2 polarization and cholesterol efflux, showing benefits in models of atherosclerosis and metabolic disease (67). Natural compounds can modulate these pathways; for example, procyanidin B2 activates PPARγ to induce M2 polarization and enhance cholesterol efflux (27), and nimbolide alleviates intervertebral disc degeneration by activating SIRT1, which in turn promotes cholesterol efflux and inhibits NF-κB/MAPK signaling in macrophages (101, 102).

### 6.2 miRNA targeting

Targeting microRNAs has garnered significant interest as a highly specific therapeutic strategy (103–105). Inhibition of miR-33 with antisense oligonucleotides (anti-miR-33) has been extensively studied (106). Anti-miR-33 therapy not only derepresses ABCA1/ABCG1 to enhance cholesterol efflux but also reprograms macrophage metabolism by boosting mitochondrial oxidative phosphorylation, restoring lysosomal function, promoting an M2-like phenotype, and even inducing regulatory T cells, thereby reducing atherosclerosis and plaque inflammation (69, 107). Similarly, inhibition of miR-34a promotes atherosclerosis regression and reverses diet-induced metabolic disorders (70). Delivery and off-target concerns, however, remain unresolved (103–107).

### 6.3 Efflux enhancement

Enhancing HDL function and cholesterol efflux capacity is another major avenue (108). Reconstituted HDL (rHDL) infusions have been shown to improve features of diabetes-accelerated atherosclerosis by reducing plasma lipids, improving glucose homeostasis, increasing vascular ABCA1/ABCG1 expression, and promoting M2 macrophage polarization (109, 110). HDL itself inhibits M1 macrophage polarization through mechanisms involving cholesterol efflux and redistribution of caveolin-1 (111). Innovative nanomedicine approaches are being developed to mimic and enhance this function (112–114). For instance, a supramolecular copolymer-modified, statin-loaded discoidal rHDL has been designed to respond to the ROS-rich plaque microenvironment, synergistically promoting cholesterol efflux and delivering an anti-inflammatory drug (atorvastatin) to foam cells, resulting in a dramatic shift from M1 to M2 polarization (115).

### 6.4 Organelle restoration

A growing body of evidence suggests that directly targeting the mitochondria-lysosome axis may offer therapeutic benefits that extend beyond simply modulating polarization markers (116–118). Enhancing autophagy and mitophagy represents a promising approach. Non-lethal sonodynamic therapy can facilitate the M1-to-M2 transition in advanced plaques by activating a ROS-AMPK-mTORC1-autophagy pathway, enhancing cholesterol efflux (119). Pharmacological activation of SIRT1, as seen with nimbolide, promotes mitophagy and restores mitochondrial function (120). Restoring lysosomal function is another critical avenue (121–124). Strategies that enhance lysosomal acidification or upregulate lysosomal hydrolase activity could improve the processing of internalized lipids, preventing the accumulation that drives foam cell formation (40, 125–127). Non-lethal sonodynamic therapy facilitates M1-to-M2 transition in advanced plaques by activating a ROS-AMPK-mTORC1-autophagy pathway, enhancing cholesterol efflux (125). While specific lysosome-targeted therapies for macrophage-driven diseases are still in early development, they represent an exciting frontier. Targeting organellar contact sites—the physical interfaces between mitochondria and lysosomes where lipid transfer and metabolic signaling occur—is a newly emerging concept (116, 128).

### 6.5 Other strategies

The sphingosine 1-phosphate receptor 1 (S1P1) agonist KRP203 polarizes macrophages toward an anti-atherogenic phenotype, enhancing efflux and efferocytosis (129). The ketone body 3-hydroxybutyrate ameliorates atherosclerosis by acting on macrophage Gpr109a to inhibit NLRP3 inflammasome activation and promote cholesterol efflux (130). Finally, cell-based therapies are being explored. Ex vivo-expanded human regulatory T cells (Tregs) can modulate macrophage responses to oxLDL, reducing inflammation and lipid accumulation (131, 132). Tregs appear to transfer cAMP to macrophages via gap junctions, enhancing ABCA1-mediated cholesterol efflux and paraoxonase-1 expression, highlighting the potential of exploiting intercellular communication to restore metabolic-immune balance.

### 6.6 Taken together

The diversity of these approaches reflects the complexity of the polarization-efflux axis. One insight emerging from this framework is that the most effective therapies may ultimately be those that simultaneously restore mitochondrial and lysosomal function—rather than those that merely shift polarization markers. Major therapeutic modalities are summarized in **Table 2**.

**Table 2:** Therapeutic strategies targeting the macrophage polarization–efflux coupling axis.

Strategy	Representative agent/method	Mechanism of action	Advantages & challenges
LXR agonist	R211945, GW3965	Activates LXR → ABCA1/ABCG1↑ + Anti-inflammatory	Potent anti-atherosclerotic, but hepatic steatosis + hypertriglyceridemia limit use
PPARγ agonist	Thiazolidinediones (TZDs)	Activates PPARγ → ABCA1/ABCG1↑ + M2 polarization + FAO↑	Improves metabolism, but weight gain, fluid retention
Combination therapy	LXR agonist + Notch inhibitor	Enhances anti-atherosclerotic effects, reduces hepatic steatosis	Effective preclinically, safety needs validation
anti-miR-33	Antisense oligonucleotide (ASO)	Derepresses ABCA1/ABCG1 + enhances FAO + promotes M2 + induces Tregs	Multi-target benefits, delivery and off-target concerns
anti-miR-34a	ASO or antagonist	Derepresses ABCA1/ABCG1 → promotes efflux	Reverses metabolic disorders, early development stage
HDL mimetic	rHDL infusion, nano-rHDL	Enhances cholesterol efflux + promotes M2 polarization + anti-inflammatory	Clinically tested, cost and administration optimization needed
Autophagy/mitophagy targeting	Sonodynamic therapy, SIRT1 agonist (nimbolide)	Activates ROS-AMPK-mTORC1-autophagy pathway	Restores organellar function, requires tissue-specific delivery
Lysosome targeting	Acidic nanoparticles, hydrolase supplementation	Restores lysosomal acidification and lipid processing capacity	Emerging approach, lacks clinical data
Cell therapy	Tregs, CAR-Treg	Transfers cAMP via gap junctions → ABCA1↑ + anti-inflammatory	

## 7 Controversies and unresolved questions

Like any developing field, the study of macrophage polarization and cholesterol efflux faces a number of unresolved questions and active debates. Several of these controversies warrant particular attention as they have implications for how the polarization-efflux coupling axis is understood and therapeutically targeted.

First, is M2 polarization always associated with high cholesterol efflux? In human atherosclerotic plaques, alternative macrophages (often labeled as M2-like) have been found to display low cholesterol handling despite high phagocytic capacity, which challenges the simple assumption that M2 equals high efflux (35). This finding raises the possibility of tissue- or stage-specific uncoupling of the axis.

Second, can LXR agonists be safely used in humans? Although LXR activation potently promotes ABCA1 expression and suppresses inflammation, clinical development has been limited by on-target side effects including hepatic steatosis and hypertriglyceridemia (66). Whether selective LXR modulators or tissue-specific targeting strategies can overcome these limitations remains an open question.

Third, do mouse and human macrophages use the same metabolic wiring? Human M2 polarization does not strictly require fatty acid oxidation, in contrast to murine cells (34). Expression of certain miRNAs, such as miR-33b, also differs between species. These observations urge caution when translating metabolism-targeting therapies from mouse models to human patients.

Fourth, can anti-miRNA therapies safely reach macrophages *in vivo*? While anti-miR-33 and anti-miR-34a have shown remarkable efficacy in mouse models, several hurdles remain unresolved for clinical translation, including efficient delivery to plaque macrophages, off-target effects, long-term metabolic consequences, and potential immunogenicity (103–107).

Addressing these controversies will likely shape the direction of research in the coming years. Among them, resolving the apparent disconnect between mouse and human macrophage metabolism appears to be a particularly high priority for the field.

## 8 Emerging frontiers in macrophage immunometabolism

Several emerging areas of investigation are poised to advance our understanding of the polarization–efflux coupling axis and may open new therapeutic avenues in the coming years.

First, single-cell macrophage states. Recent advances in single-cell RNA sequencing and spatial transcriptomics have revealed that macrophage populations within atherosclerotic plaques, diabetic kidneys, and tumors exhibit far greater heterogeneity than the traditional M1/M2 classification would suggest (6, 7). These technologies have identified disease-relevant subsets with unique metabolic and transcriptional signatures, including lipid-associated macrophages (LAMs) and TREM2-high populations. Mapping the precise polarization–efflux coupling status of these subsets in human tissues will be an important goal for developing precision immunometabolic therapies.

Second, organelle contact sites. Beyond the independent functions of mitochondria and lysosomes, the physical interfaces between these organelles—known as mitochondria-lysosome contact sites (MLCS)—are increasingly recognized as hubs for lipid transfer, Ca²^+^ signaling, and metabolic crosstalk (116, 128). These contacts regulate autophagosome formation, mitochondrial fission, and lipid flux. It is conceivable that modulating MLCS could restore the coordination of lipid influx, processing, and efflux without directly altering polarization markers, representing a conceptually novel therapeutic strategy.

Third, metabolic checkpoints (succinate, itaconate). Emerging evidence indicates that specific metabolites act as signaling molecules that control macrophage polarization. Succinate, which accumulates during M1 activation, stabilizes HIF-1α and drives IL-1β production via the succinate receptor SUCNR1. In contrast, itaconate, produced by the enzyme IRG1, exerts anti-inflammatory effects by inhibiting NLRP3 inflammasome activation and promoting NRF2-dependent antioxidant responses. These metabolic checkpoints appear to intersect directly with cholesterol efflux pathways and the mitochondria-lysosome axis (68, 69, 102, 119). Targeting succinate or itaconate metabolism could thus potentially reprogram inflammatory output while restoring lipid handling.

Fourth, lipid trafficking. The intracellular journey of cholesterol—from uptake via scavenger receptors, to processing within lysosomes, to transport through the ER and Golgi, and ultimately to efflux via ABCA1/ABCG1—involves multiple organellar compartments. Key trafficking proteins such as ORP2, StAR, and NPC1/2 regulate these steps (15, 56). Failure at any of these nodes can lead to lipid accumulation and foam cell formation. Understanding how these trafficking pathways become disrupted under chronic inflammatory stress—and how they integrate with mitochondrial-lysosomal homeostasis—is likely to uncover new therapeutic entry points.

These frontiers share a common theme: a shift from studying static correlates toward understanding dynamic, spatially organized mechanisms. They also point toward a common therapeutic logic—namely, restoring coordination within the system rather than blocking individual molecular players.

## 9 Future directions

Several key questions remain to be addressed in future research.

Can organellar dysfunction be therapeutically reversed? Directly targeting mitophagy, lysosomal acidification, or mitochondria-lysosome contact sites (MLCS) represents a promising but as yet largely unexplored frontier (116, 128). Whether such strategies can effectively restore organellar function *in vivo* and whether this restoration translates into meaningful therapeutic benefit will require systematic investigation.

How stable is trained immunity in human macrophages? A critical question is whether pro-inflammatory epigenetic memory induced by oxLDL or hyperglycemia can be erased (80, 81). If so, what strategies—such as histone demethylase activators or HDAC inhibitors—might achieve this without causing unintended effects on other cell types?

What are disease-specific macrophage states in humans? Single-cell RNA sequencing and spatial transcriptomics are expected to map the heterogeneity of macrophage populations within human atherosclerotic plaques and diabetic kidneys, thereby identifying disease-relevant pathogenic subsets (6). Determining how these subsets differ in their polarization–efflux coupling status will be an important step toward precision immunometabolic therapies.

Can targeting organelle contact sites improve outcomes? Modulating the physical interfaces between mitochondria and lysosomes (MLCS) is a conceptually novel approach that could potentially coordinate the functions of both organelles simultaneously (116, 128). Whether this can be achieved pharmacologically and whether it yields therapeutic effects beyond those of targeting individual organelles remain to be determined.

Translation of pre-clinical strategies. Finally, translating promising pre-clinical strategies—especially miRNA inhibitors and advanced nanotherapeutics—into safe and effective clinical regimens remains a significant hurdle. Delivery specificity, off-target effects, long-term safety, and the potential need for patient stratification based on inflammatory and metabolic profiles are all issues that will need to be addressed.

## 10 Conclusion

In summary, this review highlights macrophage polarization–cholesterol efflux coupling as an integrated immunometabolic framework for understanding macrophage dysfunction in chronic inflammatory disease. Rather than representing independent processes, macrophage phenotypic plasticity, intracellular cholesterol handling, and organelle homeostasis appear to be closely interconnected determinants of cellular adaptation to metabolic and inflammatory stress. Progressive disruption of this coordinated network contributes to maladaptive macrophage reprogramming, impaired resolution responses, and sustained tissue injury across diverse pathological contexts.

Importantly, this framework provides a conceptual basis for moving beyond the traditional binary polarization model toward a more dynamic systems-level understanding of macrophage functional states. However, several critical questions remain unresolved, including how organelle-specific stress signals are temporally integrated during macrophage state transitions, whether polarization–efflux decoupling exhibits disease-specific molecular signatures, and how these processes can be quantitatively resolved *in vivo*.

Addressing these challenges will require the integration of emerging approaches, including single-cell multi-omics, spatial metabolomics, organelle-resolved imaging, and computational systems modeling. Such efforts may enable precise characterization of macrophage immunometabolic states and facilitate identification of context-dependent therapeutic vulnerabilities.

Ultimately, restoring coordinated macrophage organelle function and cholesterol efflux capacity may represent a promising strategy for interrupting maladaptive inflammatory circuits and promoting tissue homeostasis across chronic inflammatory diseases.
